# Depression Following Acute Coronary Syndrome: A Review

**DOI:** 10.31083/j.rcm2409247

**Published:** 2023-09-05

**Authors:** Roubai Pan, Qin Fan, Rong Tao

**Affiliations:** ^1^Department of Cardiovascular Medicine, Ruijin Hospital, Shanghai Jiaotong University School of Medicine, 200025 Shanghai, China

**Keywords:** acute coronary syndrome, depression, prognosis, management

## Abstract

Depression is common among patients with acute coronary syndrome (ACS). Although multiple studies have confirmed that depression is an independent risk factor for poor outcomes in ACS, general awareness of this issue is still limited. Ongoing research has described detailed aspects of depression in ACS, with various mechanistic hypotheses put forward to explain the complexity of this comorbidity. Several investigations have explored management strategies in this subgroup of patients, including screening for depression, antidepressant treatment, and cardiac rehabilitation. However, evidence of long-term improvement in clinical outcomes is still scarce, and a more comprehensive understanding of the underlying mechanisms that link depression with ACS is required to further improve disease management.

## 1. Introduction

Interest in the role of depression in acute coronary syndrome (ACS) has surged in recent years because depression is recognized to be a major cause of mortality, disability and quality of life impairment [1, 2]. Depression is a frequent complication of ACS, with a literature review by Thombs *et al*. [3] finding that major depression was present in 19.8% of patients hospitalized for recent acute myocardial infarction (AMI), as assessed by a structured clinical interview. Depending on the questionnaire and rating scale used, the prevalence of significant depressive symptoms ranged from 7.3% to 31.1%. A recent meta-analysis found the pooled prevalence of depression among patients with myocardial infarction (MI) was 28.7% [4].

Several studies have consistently shown that patients with ACS and depression have a higher risk of recurrent cardiovascular events and mortality [5], poorer quality of life [6], and higher health care costs [7]. Some studies have tried to evaluate the complexity of comorbidities from the perspective of cardiac disease severity or other ACS risk factors [8, 9]. Other studies have focused on the nature of depression and found that different aspects of depression, such as specific symptoms [10] or the time of onset [11], were associated with adverse outcomes in ACS patients.

Various biological and behavioral mechanisms have been proposed to explain the relationship between depression and ACS, including inflammation [12], autonomic dysfunction [13], platelet reactivity, endothelial function [14], neuroendocrine disturbances [15], lifestyle factors, and other unmodifiable risk factors [16]. A complex interplay between these systems is likely to modulate cardiac and neuropsychiatric functions.

The role of depression in patients with ACS has generally been overlooked over the past few decades. According to a survey conducted in 2006, only half of all cardiovascular physicians considered that depression was a risk factor for coronary artery disease (CAD) [16]. With accumulating evidence of a correlation between depression and ACS, the American Heart Association (AHA) declared in 2014 that depression was an independent risk factor for ACS. This was based on an extensive review of 53 publications and four meta-analyses [17]. However, interpretation of the prognostic association between depression and ACS remains challenging due to the heterogeneity of these studies in terms of the demographic characteristics of the study sample, the definition and measurement instruments for depression, and the follow-up periods. Experts have developed several guidelines for the screening and treatment of depression in patients with coronary heart disease (CHD) [18]. Nevertheless, a cross-sectional study found that patients with these two conditions were inadequately managed for their depression by physicians, with just 6.6% being offered psychotherapy and 4.1% offered medication [19]. One possible explanation for the poor adherence to guidelines was a lack of awareness at the provider level of the connection between depression and ACS [20]. Here, we comprehensively review the correlation between depression and ACS with the aim of raising awareness of the comorbidity of these two diseases. We also summarize the possible mechanisms for this complex biobehavioral pathway, and provide further details on the robust correlation between ACS and depression. In addition, we examine the consistency of results between various studies, and discuss the optimal management strategy for this important subgroup of patients with ACS.

## 2. Depression as a Risk Factor for ACS

### 2.1 Depression and Cardiac Disease Severity in Patients with ACS

Left ventricular dysfunction is one of the most important predictors of mortality in patients with ACS. In the Myocardial Infarction and Depression-Intervention Trial (MIND-IT), a dose-response-like relationship was observed between the severity of depressive symptoms and left ventricular dysfunction in patients with AMI. This should be considered when evaluating the prognostic impact of depression on clinical outcomes from ACS [8]. Depression appears to reflect the severity of ACS. Meijer *et al*. [5] conducted the largest meta-analysis to date that examined depression as a risk factor for adverse medical outcomes in patients with ACS. These workers identified 29 studies that included 16,889 patients with MI. After a 24-month follow-up period, the pooled odds ratios (ORs) for all-cause mortality, cardiac mortality, and fatal or non-fatal cardiac events were 2.25 [95% confidence interval [CI], 1.73–2.93; *p* < 0.001], 2.71 [95% CI, 1.68–4.36; *p* < 0.001], and 1.59 [95% CI, 1.37–1.85; *p* < 0.001], respectively [5]. These effects were attenuated after adjusting for cardiac disease severity, but remained significant. An individual patient data meta-analysis was also conducted to determine whether cardiac disease severity was a confounding factor for clinical outcomes. This included 10,175 post-infarction patients from 16 studies. The hazard ratio (HR) for all-cause mortality during an average of 3.2-years follow-up was 1.32 [95% CI, 1.26–1.38; *p* < 0.001] and for cardiovascular events it was 1.19 [95% CI, 1.14–1.24; *p* < 0.001] [21]. Cox regression analysis revealed the HR for all-cause mortality was reduced by 28% and for cardiovascular events by 25% after adjusting for Killip class, left ventricular ejection fraction (LVEF), history of MI, diabetes, smoking, and body mass index. Consistent with a previous meta-analysis, depression remained independently associated with cardiac prognosis [5]. Cardiac disease severity accounted for a portion of the effects observed in these studies. In contrast, depression was negatively associated with ventricular dysfunction and troponin levels in a study of young patients with ACS [22]. Therefore, depression by itself may affect cardiac outcomes, independently of cardiac disease.

### 2.2 Depression and Cardiac Risk Factors in ACS

The role of gender has been a major research topic in ACS in recent years. The incidence of hospitalization of young women due to ACS has increased over the past two decades [23]. In addition, the mortality rate from ACS is higher in elderly women than in men, and depression is almost twice as common in women than in men [24]. In the Variation in Recovery: Role of Gender on Outcomes of Young AMI Patients (VIRGO) study, young women (<55 years) with AMI showed 60% greater odds of significant depressive symptoms than young men [25]. Depression also increased the risk of death in young women with ACS [26]. ACS was associated with greater psychological stress in women than men, as well as with depression and poor recovery [27]. A unique relationship between depressive and repolarization abnormalities was identified in women after ACS and may reflect abnormal sympathetic activation [28]. Greater sympathetic activation following ACS might contribute to this vulnerability [29]. One study demonstrated a link between limbic systems and ventricular dysfunction uniquely in women, which could partially explain the poor outcomes [30]. Ethnicity is another non-modifiable risk factor for ACS. Black race is associated with a higher risk of cardiovascular disease (CVD) mortality, with these patients also more likely to experience depressive symptoms and to remain undiagnosed [31]. Black patients with these two conditions tend to have lower socioeconomic status and a higher prevalence of lifestyle, psychosocial and clinical risk factors. These patients comprise a subgroup with an elevated risk for ACS. Another study found that depressive symptoms were related to ischemic heart disease mortality in elderly black patients over a 12-year follow-up period [32]. However, further evidence is needed as there are still very few studies on this topic.

Patients with advanced age have a high risk of mortality from ACS, which may be explained by a higher prevalence of comorbid diseases and delayed presentation to hospital [33]. Late-life depression refers to depressive syndrome in adults >65 years [34]. ACS is an underlying condition that usually occurs in the context of other medical disorders [35]. Late-life depression is often accompanied by cognitive impairments and ischemic brain lesions, which increase the risk of death in patients with ACS [36]. It has been reported that older patients with ACS and depression have an almost four-fold higher risk of dying within the first 4 months after discharge [37]. They were prescribed fewer medications for ACS and had difficulty following the recommendations to reduce cardiac risk. This finding highlights the importance of screening for depression in the elderly patient subgroup and the need for active treatments.

Diabetes mellitus is another strong predictor of recurrent ischemic events and mortality in patients with ACS [38]. Depression is present in approximately one in five adults with type 2 diabetes and is associated with increased risks of mortality, work absenteeism, poor disease management, and poor health outcomes [39]. Depression and diabetes share common mechanisms including inflammation, neuroendocrine dysfunction, and insulin resistance [40]. These play vital roles in the progression of macro- and microvascular lesions in patients with ACS.

There is a phenomenon known as the obesity paradox in ACS patients, whereby low-weight and overweight patients with ACS have a higher risk of mortality than normal-weight patients [41]. This paradox might be explained by follow-up time and obesity indices. In the long-term, the protective effect of obesity is no longer present or may even be reversed. When using waist circumference to evaluate central obesity, a greater waist circumference was associated with adverse outcomes [42]. Depression and obesity frequently co-occur and have a reciprocal relationship [43]. A recent study reported that obesity was predictive of depression after ACS [44], with the association being stronger in patients with central obesity [45]. Several hypotheses have been proposed to explain this relationship. Depression, obesity and ACS are all associated with lifestyle risk factors, such as poor diet and physical inactivity [46]. Biological pathways such as inflammation and hyperactivation of the hypothalamic-pituitary-adrenal (HPA) axis might be involved in the co-occurrence of these conditions. However, in the Enhancing Recovery in Coronary Heart Disease patients (ENRICHD) study [47], no interaction was found between weight change and depression in patients with ACS. More studies are therefore required to fully elucidate this relationship.

### 2.3 Depression Measurement Instruments and Depressive Symptoms in Patients with ACS

Unlike other diseases that can be diagnosed using imaging techniques or serum biomarkers, the diagnosis of depression relies mainly on evaluation of clinical symptoms. This means that various measurement instruments used in different studies could influence the apparent prognostic effects of depression. The most widely used self-reported depression scale for studying the association between ACS and depression is the Beck Depression Inventory (BDI). This is available in different versions including BDI-1A, BDI-II, and BDI-Fast Screen (BDI-FS). Other commonly used self-report questionnaires include the Hospital Anxiety and Depression Scale (HADS) [48], the Patient Health Questionnaire (PHQ) [49], and the Zung Self-Rating Depression Scale [50]. A cut-off score is used to distinguish patients who present with “clinically significant” depression. In some studies, patients with abnormal screening results underwent additional psychiatric evaluation to diagnose major depressive disorder using a standardized psychiatric interview by trained interviewers. Interviews were conducted based on the standard criteria outlined in the Diagnostic and Statistical Manual of Mental Disorders, or the International Classification of Diseases. Not all patients classified as having clinically significant depression met the criteria for major depression. In a systematic review of the accuracy of depression-screening instruments in ACS [51], BDI-II displayed an overall sensitivity of 90% [95% CI, 86%–92%] and specificity of 80% [95% CI, 68%–88%] compared with a validated criterion standard. Both these sensitivity and specificity results were deemed acceptable. Brief approaches, such as the selection of just one or two items from traditional screening tools, may have consistent diagnostic accuracy metrics with longer screens and are easier to apply in clinical practice. In secondary analyses of the meta-analysis mentioned above [6], all-cause mortality was different between studies that used interview-based instruments or self-report instruments [OR 3.69 for interview-based instruments, 95% CI, 2.05–6.63;* p* < 0.001 vs OR 1.83 for self-report instruments, 95% CI, 1.51–2.23; *p* < 0.001]. These findings indicate that the type of instrument used can affect the prognostic value, and thus structured interviews are needed for ACS patients with depressive symptoms. Efforts have been made to evaluate the internal consistency between screening scales in the context of CAD. However, the performance of each instrument was found to vary in different studies, possibly also because of differences in the target group of patients [48, 52].

Much interest has been focused on specific depressive symptoms and cardiac prognoses. Studies on the association between depressive symptoms and ACS have often divided depressive symptoms into three dimensions: somatic, cognitive, and affective. Somatic symptoms of depression can be defined as bodily sensations of concern, such as sleep and appetite changes, fatigue, shortness of breath, and pain. Cognitive symptoms include deficits in executive function, attention, short-term memory, and psychomotor skills. Affective symptoms are related to emotional symptoms such as depressed mood and anhedonia. In the MIND-IT study [53], researchers created a new dimensional structure of depressive symptoms in patients with MI by combining explorative and confirmatory factor analyses of the BDI items. They distinguished three factors (somatic/affective, cognitive/affective, and appetitive) and categorized the symptoms in BDI items according to these factors. For example, fatigue is associated with both somatic and affective factors, and was therefore defined as a somatic/affective symptom in the study. The authors found that somatic/affective symptoms were correlated with cardiovascular mortality and cardiac events after an average of 2.5-years of follow-up. This association was partially confounded by cardiac health status, but remained significant after adjustment. Conversely, cognitive/affective symptoms were negatively associated with cardiovascular death in multivariate analyses. Researchers in the ENRICHD clinical trial used the same analytical method, with the symptoms divided into two dimensions of somatic and cognitive [10]. In this study, somatic symptoms at 12 months post-MI predicted outcomes, but not at baseline. However, other studies reported that cognitive symptoms were correlated with a high risk of cardiac mortality [54, 55]. A meta-analysis that investigated the association between specific depressive symptoms and cardiovascular prognosis found that both somatic/affective and cognitive/affective symptoms were associated with cardiac prognosis [56]. The correlation between somatic/affective factors and cardiac prognosis was stronger, as the association remained significant in fully adjusted analyses. Somatic symptoms are related to autonomic dysfunction and neuroendocrine disturbances, which are the underlying mechanisms in the vicious cycle of depression and ACS [57]. These studies suggest that treatment of depression in patients with ACS should target somatic symptoms to improve cardiac prognosis. There are several methodological inconsistencies between studies, such as in the classification of symptoms. For example, sadness was defined as a somatic/affective symptom in MIND-IT, but as a cognitive symptom in the ENRICHD study. Thus, the superiority of specific symptoms for cardiac prognosis remains inconclusive [58]. Sleep disturbance is a common somatic symptom [59]. In the Escitalopram for DEPression in ACS (EsDEPACS) study [60], sleep disturbance during the acute phase of ACS increased long-term all-cause mortality in patients with ACS [HR 1.08–1.59]. Further trials are warranted on the interventions needed to improve sleep disturbances and their effect on cardiac outcomes.

### 2.4 Onset and Prognosis of Depression in Patients with ACS

Emerging studies suggest that the association between depression and cardiac outcomes in patients with ACS may depend on the time of onset of depression. Patients with ACS and depression were categorized into two groups according to the sequence of events. One group without a history of depression and constituting nearly half of all cases experienced their first depressive episode at the time of ACS [61, 62]. The second group had a history of depression before ACS and experienced ongoing or recurrent depression after ACS. In the Depression After Myocardial Infarction (DepreMI) study, only incident, post-MI depression was associated with cardiovascular prognosis [11]. Another study also compared these two groups. First-ever post-MI depression was related to cardiac function, revascularization during hospitalization, and arrhythmic events, which may be triggered by severe MI [63]. Ongoing or recurent depression was associated with neuroticism and exacerbated depression before MI. Identification of the different subtypes could help to formulate the appropriate treatment strategies. First-ever post-MI depression may require a treatment strategy that targets the consequences of MI, rather than a purely depression-oriented treatment. However, other studies have suggested that patients with ACS who experienced recurrent depression were at a particularly high risk of cardiac death [64]. In a nationwide, population-based cohort study [65], patients with MI and a previous diagnosis of depression had a higher mortality risk than those without previous depression. A meta-analysis aimed at evaluating the timing of depression and cardiac prognosis did not reach any firm conclusion due to inconsistent findings [66]. This inconsistency may have derived from selection bias, study quality, definition of incidence periods in various studies, and recall bias [66]. More solid evidence is needed to establish the prognostic significance of depression onset before or after ACS.

One study evaluated the onset of depression at different times following ACS [67]. Depression within 2 weeks or 1-year after ACS was associated with adverse outcomes. The patient subgroup with depression at both the baseline and at 1-year follow-up had the highest risk of mortality from cardiac events. This finding indicates that screening for depression should be recommended during both the early and late phases of ACS, and that more attention should be given to patients with persistent depression.

## 3. Potential Mechanisms Mediating the Effect of Depression on ACS

### 3.1 Inflammation

Several studies have demonstrated the effects of inflammation on the prognosis of ACS. Inflammation participates in cardiac repair after ACS, the severity of which can affect cardiac function and events [68]. It contributes to plaque instability and predisposes patients to recurrent atherothrombotic events [69, 70]. Various inflammatory biomarkers are elevated in ACS and have been linked to poor cardiac outcomes, including C-reactive protein (CRP) [71], interleukin-6 [72], and tumor necrosis factor-α [73]. Dysregulated inflammatory responses are known to occur in depression and account for the observed comorbidity with other diseases [74]. Lespérance *et al*. [75] reported that depressed patients have higher CRP levels than non-depressed patients. An interaction occurs between CRP levels and BDI scores in the prediction of major adverse cardiac events (MACE) in patients with ACS. The overlapping prognostic value indicates the effect of depression on ACS may be partly mediated by inflammation [12]. Other inflammation markers such as interleukin-17 and tumor necrosis factor-α were also associated with depressive symptoms and MACE in patients with ACS [76]. Statin use in patients with ACS decreases both inflammation and depressive symptoms, thus supporting the inflammatory hypothesis as underlying the comorbidity [77]. Conversely, some studies have reported no difference in the inflammatory status between depressed and non-depressed patients with ACS [78, 79]. In these patient cohorts, inflammation could not explain the association between depression and cardiac events. Inflammatory markers in the plasma change over time and may vary with different phases of ACS, as well as with depression status. Most studies have been based on the evaluation of inflammatory markers and depression status at a single time point. Therefore, a cause-effect relationship between inflammation and comorbidity has yet to be proven.

### 3.2 Cardiac Autonomic Dysfunction

Ischemia directly damages the cardiac autonomic nerves in ACS. Consequently, an imbalance of sympathetic and parasympathetic activity occurs to preserve hemodynamic changes [80]. As the stimuli persist, the functional and structural maladaptation in the cardiac autonomic nervous system (ANS) worsens and confers additional risks of cardiac events and mortality. Alterations in heart rate are easily measured clinically from electrocardiogram recordings and are used to quantify cardiac autonomic modulations, such as heart rate variability (HRV) [81]. HRV is defined as beat-to-beat variations in the heart rate [82]. In response to environmental changes, the central nervous system receives signals through neuroception and activates specific components of the vagal system via neural circuits, resulting in different functional changes in the somatomotor and visceromotor cortices [83]. HRV is the result of adaptation of the ANS to environmental changes, which may be impaired in psychiatric disorders. Thus, HRV may be used as a warning sign of psychiatric disorders, while also serving as “a bridge” between the heart and brain. Frequency-domain methods are used to study HRV by dividing the heart rate signal into its constituents (frequencies) and quantifying their relative intensities (power). High-frequency HRV primarily reflects parasympathetic vagal activity, while low-frequency (LF) HRV is complex and may have both sympathetic and parasympathetic influences. The long periods of rhythm and circadian, neuroendocrine rhythm are reflected by very low frequency (VLF) and ultra-low frequency HRV, respectively. Other HRV metrics include the time-domain index (a method to quantify variations within a specific time) and non-linear index (a method to quantify the irregularity and unpredictability of the heart rate based on chaos theory) [84]. The HRV is low during sympathetic activation and high during parasympathetic activation. According to the Framingham Heart Study, reduced HRV is an independent prognostic factor for adverse cardiac events [85]. A recent meta-analysis found that HRV was lower in patients with depression compared with healthy controls [86]. In the ENRICHD clinical trial [13], the four indices of HRV measured using the frequency domain method were significantly lower in patients who developed depression following ACS. The HR for all-cause mortality [HR 2.8, 95% CI, 1.4–5.4; *p* < 0.003] fell by almost a quarter after adjustment for VLF [HR 2.1, 95% CI, 1.1–4.2; *p* = 0.03] [87]. Premature ventricular contractions (VPCs) are associated with depression in cases of cardiac mortality [88]. The heart rate first accelerates and then decelerates after the VPC. This response pattern is thought to be regulated by the baroreceptor reflexes and the parasympathetic nervous system. Heart rate turbulence indices, including the turbulence onset and slope, are used to quantify the heart rate response to VPCs [89]. When heart rate turbulence and VLF were added to the model, the HR decreased further to 1.6 [95% CI, 0.8–3.4; *p* = 0.18]. The combination of VLF and heart rate turbulence accounts for approximately half of the effect of depression on the survival of these patients. In the Sertraline Antidepressant Heart Attack Randomized Trial (SADHART) [90], the differences in HRV became larger in depressed patients at 16 weeks after ACS onset, suggesting that HRV recovery was impaired in these patients [90]. Some authors have reported a potential link between cardiac autonomic dysfunction and inflammation in patients with ACS [91, 92]. This supports the notion that a network of biological variables mediates the relationship between ACS and depression.

### 3.3 Platelet and Endothelial Dysfunction

Platelets play a critical role in ACS and are associated with recurrent thrombotic events after ACS. Increasing evidence shows that many functional aspects of platelets, including platelet activation and aggregation, affect the comorbidity of ACS and depression. Platelet function was reported to be hyperactive in patients with ACS and depression [93]. Some important signaling pathways for platelet aggregation, such as serotonin and adenosine diphosphate signaling, were altered in these patients and linked to the occurrence of cardiac events [93, 94]. However, inconsistencies exist in the alteration of indices among these studies. Currently, there are no well-established methods for assessing platelet function. Furthermore, studies that investigate specific platelet indices as risk factors for the association between ACS and depression are lacking. As more methods are developed for assessing platelet function, the role of platelet dysfunction in the association between depression and ACS can be further elucidated.

Because the endothelium is essential for vasodilatation, anticoagulation and fibrinolysis, endothelial dysfunction is closely related to the pathophysiology of ACS [95]. The most commonly used method for evaluating endothelial function is flow-mediated dilatation (FMD). An increase in flow is induced by the inflation and subsequent release of a sphygmomanometer cuff on the distal forearm, and the impact of this ‘physiological’ stimulus on artery diameter above the elbow was assessed by high resolution ultrasound. FMD reflects endothelial-dependent vasodilatation, which depends on the bioavailability of local nitric oxide [96]. In a recent meta-analysis, patients with depression were found to have lower FMD [97]. In a study involving patients with CHD [98], FMD was significantly impaired in depressed patients compared to non-depressed patients [adjusted mean ± SE: 4.36 ± 0.75% vs 7.46 ± 0.89%, *p* = 0.001]. However, no direct evidence exists for an association between impaired endothelial function and cardiac outcomes in patients with ACS and depression. Some studies have found that endothelial progenitor cells, which are the precursors for vascular endothelial cells during vessel repair, play an important role in both depression and ACS [99, 100]. The number of endothelial progenitor cells was decreased in patients with ACS and depression, and this factor may also serve as a predictor of the severity of ACS in patients with depression [101]. Possible mechanisms could involve endothelial function, platelet function, and inflammation, although this requires further clarification [102, 103].

### 3.4 Dysfunction in the Hypothalamic-Pituitary-Adrenal (HPA) Axis

Dysfunction of the HPA axis has been implicated in the pathophysiology of depression. An increase in plasma cortisol level is the most consistent finding in depression. It results from excessive cortisol release induced by stress and impaired glucocorticoid receptor-mediated feedback inhibition [24]. Hyperactivity of the HPA is associated with hypertension and hyperglycemia, which in turn increase the risk of CVD. A recent study found that high cortisol levels were associated with ACS severity and mortality during hospitalization [104]. Activation of the HPA axis is prolonged in patients with ACS [105], while a blunted cortisol awakening response was also observed in these patients [15]. Rather than the hyperactive HPA axis often observed in depression, a flatter cortisol rhythm was seen in patients with CAD and depression [106], indicating an increased vulnerability to inflammation. The central corticosteroid signaling pathway, which was found to be impaired in rodent models, may contribute to the poor outcome of patients with depression after MI [107]. These findings provide evidence of HPA dysfunction in patients with ACS and depression, although its effect on cardiac outcomes remains unknown.

### 3.5 Lifestyle Factors 

Depression is associated with lifestyle factors that increase the risk of ACS [108]. Smoking is more frequent in adults with depression [109], while the cessation of smoking after ACS may improve depressive symptoms [110]. Depression is also correlated with a Western-style dietary pattern characterized by elevated consumption of high-fat products and low consumption of fruit and vegetables, which further contributes to ACS [111]. Physical activity is another important aspect of lifestyle. Based on self-report questionnaires, depression was associated with low physical activity in patients with ACS [112, 113]. A study that applied objective measures to evaluate physical activity found that <20% of ACS patients met physical activity guidelines [114]. No difference was observed between depression subgroups in terms of the proportion of patients who met the recommended exercise guidelines. In the ENRICHD study, patients who exercised regularly had a lower risk of fatal events [HR, 0.62; 95% CI, 0.44–0.86; *p* = 0.004] and of non-fatal AMI [HR, 0.72; 95% CI, 0.52–0.99; *p* = 0.044] [16]. In the Heart and Soul Study [115], a 31.7% reduction was observed in the association between depressive symptoms and cardiovascular events after adjusting for physical activity, with the association no longer significant. A similar reduction in effect size was observed after substituting self-reported physical activity with an objective measure of exercise capacity using a treadmill [HR, 0.96; 95% CI, 0.70–1.31; *p* = 0.79] [115], suggesting that physical activity may be a significant contributor to poor prognosis in patients with ACS and depression. However, most prospective studies on physical activity and depression used self-reported methods to measure physical activity, which often exhibit a low correlation compared with objective measurements [116]. Participants with depressive symptoms are also more likely to under-report their physical activity. Another important issue is the difficulty of patients with ACS to maintain a healthy lifestyle. Although their lifestyle improved after the first coronary event, this usually subsided after the first year. Depression is related to maladaptive lifestyles in patients with ACS [117]. The above findings emphasize the need to strengthen healthy lifestyles in this group of patients.

Poor medication adherence is a common problem among patients with ACS and depression [118]. Depression has been linked to inadequate treatment with antiplatelet therapy, β-blockers and statins after percutaneous coronary intervention (PCI), thereby affecting long-term cardiac outcomes [119]. In a study that assessed the relationship between aspirin adherence and depression following ACS [120], improvements in depressive symptoms were found to increase the adherence rates. This study highlights the importance of early recognition and intervention for poor medication adherence in the secondary prevention of patients with ACS and depression.

### 3.6 Gut Microbiome

Several studies have shown that the gut microbiome plays a role in ACS. Alterations in gut microbiota composition can result in changes in appetite, production of inflammatory factors, and accumulation of the pro-atherogenic metabolite trimethylamine N-oxide (TMAO) [121]. These risk factors further contribute to the development of ACS. The gut microbiome is also closely associated with depression. It can modulate brain development and function through microbial metabolites and immune mediators that subsequently trigger changes in neurotransmission, neuroinflammation, and behavior [122]. Several studies have identified similar alterations in the gut microbiota in both ACS and depression. For example, the relative abundance of the phyla *Firmicutes* decreased in patients with ACS and depression, whereas *Proteobacteria *increased [123, 124]. The shift in gut microbiota composition could lead to a “leaky gut” that allows lipopolysaccharides to enter the circulation and to trigger systemic inflammatory processes [125]. Increased systemic inflammation affects the progression of both depression and ACS. Alterations in the composition of gut microbiota causes changes in gut-derived metabolites, most of which are absorbed into the blood and cause perturbations in serum metabolomic patterns. Some metabolites including uremic toxins, tryptophan and its derivatives, short-chain fatty acids and TMAO were shown to be involved in the pathophysiology of ACS and depression [126]. These findings reveal new paradigms and therapeutic directions for the comorbidity of ACS and depression.

## 4. Management of Patients with ACS and Depression

### 4.1 Screening for Depression

Despite the high prevalence and robust association between depression and increased morbidity and mortality after ACS, the utility of routine screening for depression in patients with ACS remains debatable. The AHA and the American Academy of Family Physicians both recommend regular screening for depression using validated questionnaires in patients with CHD or MI [18, 127]. The European Society of Cardiology guidelines recommend screening for depression as a risk modifier in patients at high risk of CVD [128]. The European Society of Preventive Cardiology also recommends that screening for depression be included in cardiac rehabilitation programs [129]. However, some studies have pointed out that screening for depression might not affect clinical outcomes. In a randomized clinical trial that enrolled 1500 patients with ACS [130], screening for depression did not alter quality-adjusted life-years, depression-free days, or self-harm. One explanation for this disappointing result was that screening for depression on its own without further intervention has no impact on cardiac outcomes. In the DEPACS study [131], patients with ACS who screened as depression-positive showed a higher incidence of MACE [adjusted HR, 2.15; 95% CI, 1.63–2.83] over a median 8.4-year follow-up period. Those diagnosed with depressive disorder and treated with escitalopram had the lowest incidence of composite MACE (40.9%) compared to those treated with placebo (53.6%) or those receiving medical treatment for ACS only (59.6%) [131]. Although a direct comparison was not made between the screened and non-screened groups, this study suggests that screening for depression can help to identify patients who are at high risk, as well as improving long-term cardiac outcomes. A recent study reported that approximately 60% of patients undergoing cardiac rehabilitation underwent screening for depression [132]. It is clear from this result that screening practices in routine cardiac rehabilitation are still far from optimal.

The two-step PHQ-2 is a self-report questionnaire recommended by the AHA as the first step in depression screening for patients with ACS. It includes two items (sad mood and anhedonia) with yes/no options that can be asked verbally by physicians. Patients who answer “Yes” to either question in the two-step PHQ-2 should be assessed with PHQ-9, which is a more comprehensive questionnaire that assesses each of 9 domains that define depression. Those with PHQ-9 scores >10, or who answered “Yes” to the ninth question assessing suicidal ideations, should be referred for further clinical evaluation [18]. Other tools for screening of depression, such as the BDI and HADS, measure the severity of depressive symptoms. No specific tools are recommended in other guidelines. Owing to their convenience and availability in multiple languages, PHQ-2 and PHQ-9 appear to be the best instruments for the screening of depression in patients with ACS [49]. However, patients with ACS and depression are often affected by somatic symptoms such as fatigue and insomnia, and hence emotional symptoms may not be the main complaints of these patients [133]. Such patients may be neglected when the simplified version of the questionnaire is used.

### 4.2 Revascularization and Depression

Intervention in the culprit vessels is the standard treatment strategy for ACS, with PCI considered to be one of the primary approaches. Emerging evidence suggests that depression is associated with poorer clinical outcomes in patients undergoing PCI. In a study of 1112 patients with stable angina pectoris or ACS who underwent PCI [134], depression after PCI was associated with a 77% increased risk of all-cause mortality after 10 years of follow-up [HR, 1.77; 95% CI, 1.36–2.29]. A similar effect on patients who underwent PCI was observed in a recent meta-analysis [relative risk, 1.57; 95% CI, 1.28–1.92] [135], with neither the assessment time or the follow-up time affecting the relationship. However, most studies did not report separate results according to the indications for PCI, and hence caution should be exercised when generalizing the results to patients with ACS.

Coronary artery bypass grafting (CABG) is another approach for revascularization in patients with ACS when PCI fails or the coronary occlusion is not amenable to PCI. Unlike PCI, CABG is rarely performed in emergency settings. Thus, in one study, patients who underwent CABG and had depression were divided into preoperative and postoperative depression groups [136]. In a study on postoperative depression in patients undergoing elective CABG surgery [137], depressive symptoms one year after surgery were associated with a slightly higher mortality rate over an 11-year follow-up period [adjusted HR, 1.05; 95% CI, 1.01–1.10; *p* = 0.03 for males; adjusted HR, 1.07; 95% CI, 1.02–1.13; *p* = 0.013 for females]. Other studies have focused on the association between preoperative depression and clinical outcomes in CABG patients. In a meta-analysis that included seven studies with a combined study population of 89,490 [138], patients with preoperative depression exhibited a pooled HR of 1.46 [95% CI, 1.23–1.73; *p* < 0.0001] for all-cause mortality following CABG. However, only three of these studies used questionnaires to define depression, whereas the other four used medication with antidepressants as the definition [138]. In subgroup analysis [138], patients in which depression was defined from questionnaires did not have a significantly increased risk of mortality [HR, 1.47; 95% CI, 0.94–2.31], possibly due to the relatively small number of patients in this subgroup. Patients who underwent CABG were less likely to be screened for depression than those who underwent PCI [132], suggesting that more attention should be paid to this patient group.

### 4.3 Medications for ACS and Depression

Aspirin is an antiplatelet drug recommended for ACS. In a recent meta-analysis [139], aspirin use was associated with a lower risk of depression [OR, 0.85; 95% CI, 0.75–0.97; *p* = 0.02]. Consistent with this result, poor aspirin adherence has been shown to account for a substantial proportion of the excess prognostic risk associated with depressive symptoms after ACS [140]. While the protective effect of aspirin can be attributed to its anti-inflammatory properties, its antidepressant effect is still open to debate. Evidence suggests that aspirin use in elderly individuals may increase the risk of depression [35], and aspirin use for the long-term management of late-life depression may worsen depressive symptoms [141].

Statins are used to reduce low-density lipoprotein cholesterol levels in ACS patients. In addition to lowering cholesterol, they also have the ability to reduce oxidative stress and modulate inflammation. Statin use has been associated with a reduced risk of depression [HR, 0.91; 95% CI, 0.87–0.94] [142]. In the EsDEPACS study, the use of statins and especially lipophilic statins was associated with high response rates to escitalopram therapy in patients with ACS and depression [143]. Combinations of statins and antidepressants such as selective serotonin reuptake inhibitors (SSRIs) are thought to be safe [144]. Statins appear to be a good add-on option along with standard therapy for post-ACS depression [145].

β-blockers are a family of neurohormonal agents used for secondary prevention after ACS. Although β-blockers were originally thought to cause depression, recent studies suggest they may actually lower the risk of depression. In a recent meta-analysis [146], the incidence of depression in the β-blockers group was not significantly different to that of the placebo group [OR, 1.02; 95% CI, 0.83–1.25; *p* = 0.88]. In a case-control study that included 118,705 patients with incident depression, only short-term use of β-blockers was linked to the development of depression [147]. The risk for depression was limited to propranolol users with neuropsychiatric disorders. This finding indicates that depression may be related to underlying disease, rather than to the use of β-blockers. In a multicenter study of patients with MI, no difference was seen in the incidence of depression between β-blocker and non-β-blocker users [148]. Therefore, it is reasonable to prescribe β-blockers to patients with MI and depression.

Angiotensin-converting enzyme inhibitors and angiotensin receptor blockers are believed to exert antidepressant effects by acting on the renin-angiotensin system [149]. However, more clinical trials are needed to confirm this viewpoint.

### 4.4 Depression Management in ACS Patients

Given the high prevalence and clinical implications of depression in patients with CAD, cardiologists are advised to initially manage mild-to-moderate depression. Determining whether patients with ACS and depression might obtain long-term clinical benefit from antidepressant treatment is of vital importance. Treatments for depression include antidepressant medications, psychotherapy, and exercise.

#### 4.4.1 Antidepressant Medication

SSRIs are recommended for the treatment of depression in patients with ACS. The cardiovascular safety of sertraline was demonstrated in the SADHART study, which randomized 369 patients with depression and ACS to either the sertraline or placebo group in a double-blind manner [150]. Safety outcomes including LVEF (primary outcomes), treatment-emergent increase in VPCs, and QT interval prolongation were similar in both the intervention and control groups at the 24-week follow-up, while the depressive symptoms improved in the treatment group [150]. It is worth noting that sertraline was associated with a lower incidence of severe cardiovascular events than placebo (14.5% vs 22.4%, respectively), although this did not reach statistical significance. Although this trend may indicate a cardioprotective effect of sertraline in the treatment of patients with depression [150], the study was not designed to evaluate the efficacy of sertraline on long-term cardiac outcomes. The ENRICHD randomized clinical trial investigated treatments for depression (mainly psychotherapy, as discussed in the next section) after MI. In post hoc analysis, the use of SSRIs was associated with lower all-cause mortality or recurrent MI over a 4-year follow-up period. However, since SSRIs were not designed as a primary intervention, a cause-and-effect relationship between the use of SSRIs and cardiac outcomes could not be established. The MIND-IT study investigated active treatment with antidepressant medications on long-term improvements in depression and cardiac outcomes in 2177 patients with MI [151]. The treatment modalities included antidepressant medications with noradrenergic actions and specifically, the serotonergic antidepressant mirtazapine as the first choice in a double-blind placebo-controlled fashion. Open treatment with the SSRI citalopram was used in case of refusal or insufficient treatment response. These were combined with other therapies such as psychotherapy and cardiac rehabilitation. At the 18-month follow-up, no significant differences were observed between the intervention and control groups with respect to long-term depression status (13% in the usual care arm vs 14% in the intervention arm, *p* = 0.76). Moreover, no differences in cardiac outcomes were observed between patients who did or did not receive antidepressant medication [151]. Although negative results were observed in the MIND-IT study, an association between the relief of depressive symptom burden and cardiac outcomes could not be ruled out. In a subgroup analysis [152], the incidence of cardiac events was higher (25.6%) in patients who did not respond to antidepressant medications (at least 50% reduction in the Hamilton Depression Rating scale) compared to 7.4% in responders. This observation indicates that cardiac outcomes may depend in part on improvements in depressive symptoms. The EsDEPACS study investigated the use of escitalopram for the treatment of depression following ACS [153]. A total of 300 patients were randomized to either escitalopram or a placebo group for 24 weeks. Treatment with escitalopram for 24 weeks was associated with a lower risk of composite MACE [HR, 0.69; 95% CI, 0.49–0.96; *p* = 0.03] and individual MACE after a median of 8.1 years. This is the largest study cohort to report beneficial effects of antidepressant medications on long-term cardiac outcomes. In post hoc analysis, patients with remission of their depression had lower risks of composite MACE, all-cause mortality, and PCI than those without remission. These findings concur with results from the MIND-IT study, and emphasize the need to discover new modalities to deal with treatment-resistant depression.

Recent studies have identified the SSRI paroxetine, a novel G protein-coupled receptor kinase-2 inhibitor that is capable of reversing cardiac dysfunction and remodeling in experimental models of AMI [154]. However, in randomized clinical trials paroxetine failed to improve LVEF or LVEF recovery in patients with anterior MI and impaired cardiac function [155, 156]. Despite these negative results, the effects of paroxetine-mediated G protein-coupled receptor kinase-2 inhibition on cardiac remodeling merits further research.

Another issue worth noting is the safety profile of antidepressant medications in patients with ACS. Tricyclic antidepressants and monoamine oxidase inhibitors are rarely used in patients with CVD due to cardiotoxic effects such as QT interval prolongation and hypertension [157]. Most SSRIs, especially citalopram, have the potential to cause some QT interval prolongation [157], with the risk increasing at doses higher than the recommended dose. Serotonin and norepinephrine reuptake inhibitors (SNRIs) have more adverse effects than SSRIs, including hypertension [158]. Before prescription, patients should undergo a screening electrocardiogram for baseline QT interval and later they should also be checked periodically for changes. Patients undergoing antihypertensive therapy should be monitored for blood pressure after initiation of SNRIs. SSRIs are the preferred choice for depression in patients with ACS. Drug–drug interactions between antidepressant drugs and cardiovascular drugs are common, and the combined use of SSRIs and antiplatelet therapy increases the risk of bleeding events in patients with ACS [159]. Some SSRIs, including fluoxetine and fluvoxamine, are cytochrome P450 (CYP)2C19 inhibitors. CYP2C19-inhibiting SSRI therapy in clopidogrel users was associated with a moderate risk of ischemic events [HR, 1.11; 95% CI, 1.01–1.22] [160].

Collectively, there is no solid evidence to support the view that antidepressant medications, including SSRIs, improve cardiac outcomes in patients with ACS and depression. The risk of adverse events and of drug–drug interactions must be considered when initiating pharmacotherapy in these patients.

#### 4.4.2 Psychotherapy

Psychotherapy refers to a broad range of therapeutic modalities including cognitive behavioral therapy (CBT), interpersonal psychotherapy, and positive psychology therapy (PPT). CBT is the most extensively studied intervention for patients with ACS and depression. It is characterized by the identification and modification of negative thoughts that adversely affect emotions and behaviors. The ENRICHD study is the largest investigation on the effect of psychotherapy for the treatment of depression following ACS [161]. In this study, 2481 patients with depression and perceived low social support after MI were randomized to CBT-based psychosocial intervention and the usual medical treatment groups. The composite primary endpoint of the study was mortality or recurrent MI. After an average follow-up of 29 months, no difference was observed in the primary endpoints between the intervention and control groups. Other forms of psychotherapy, such as problem-solving therapy (a form of psychotherapy to augment patients’ skills in evaluating and addressing individual psychosocial problems), PPT (enhancing well-being by developing individual strengths and positive psychological dimensions) [162], and well-being therapy [163] have shown promise for improving depressive symptoms. Some studies have tried to combine psychotherapy and media, such as telephone and the internet, for the treatment of depression following ACS [164]. In the U-CARE Heart study that enrolled 239 patients with depression following ACS, internet-based CBT was comparable with the usual treatment in reducing depressive symptoms, but showed a trend for increased risk of CVD [HR, 1.8; 95% CI, 0.96–3.4; *p* = 0.07] [165]. The main concern with internet-based psychotherapy is low treatment adherence, which was below 50% in most studies [166]. Psychotherapy was not the only intervention used in many studies so far, and the combination of psychotherapy and pharmacotherapy might result in better clinical outcomes. In the ENRICHD study, a reduction in depression severity was associated with improved survival only in those patients who received CBT and antidepressant medications [167]. In the Coronary Psychosocial Evaluation Studies trials, a combination of problem-solving therapy and antidepressant medication improved cardiac outcomes and depressive symptoms during active treatment [168]. A meta-analysis showed that CBT-based and PPT-based psychological interventions reduced the risk of cardiovascular events, MI, and angina in patients with CAD [169]. Psychotherapy may therefore be helpful for the treatment of depression following ACS.

#### 4.4.3 Exercise-Based Cardiac Rehabilitation

Exercise and cardiac rehabilitation are also effective therapies for depression when conducted in cardiac settings. Cardiac rehabilitation involves a combination of exercise, nutritional management, and lifestyle modification to improve the health of patients with CVD. Exercise-based cardiac rehabilitation has been shown to benefit patients with CHD [170]. In the Understanding the Prognostic Benefits of Exercise and Antidepressant Therapy study [171], exercise improved depressive symptoms and HRV in patients with stable CHD, and this presumably also had a favorable effect on cardiac outcomes [171]. In a study of 522 patients with CHD after a recent coronary event [172], cardiac rehabilitation (exercise, dietary intervention, health education) resulted in a 63% reduction in depressive symptoms and a 73% decrease in mortality. In addition to improving physical activity, cardiac rehabilitation helps to improve behavioral aspects related to food consumption, stress management and sleep quality [173]. A recent meta-analysis showed that exercise-based cardiac rehabilitation alleviated depressive symptoms in MI patients [174]. Exercise, or exercise-based cardiac rehabilitation, should therefore be recommended as the basic treatment for patients with CHD and depression.

#### 4.4.4 Other Potential Therapies

The gut microbiota is involved in the pathophysiology of both ACS and depression. Targeting of the gut microbiota could therefore be another potential therapy. Preliminary clinical trials have shown that supplementation with probiotics such as *Lactobacillus rhamnosus*, *Bifidobacterium lactis*, and/or prebiotics in patients with ACS improved depressive symptoms and alleviated systemic inflammation [175, 176]. These findings suggest new therapeutic directions for the treatment of depression following ACS. However, the evidence is still sparse and there is inconsistency regarding the use of different probiotics. Further research is required to confirm the efficacy and safety of probiotics. Recent studies have suggested that traditional Chinese medicine exerts antidepressant effects in animal models with ACS [177, 178]. These mechanisms may involve anti-inflammatory effects, reduction of cell apoptosis, and alteration of neuroendocrine metabolism. Xinkeshu, a well-known and patented Chinese drug, has been widely studied in patients with CHD and depression after PCI [179]. A meta-analysis showed that Xinkeshu tablets combined with conventional treatments led to improved depressive symptoms and blood lipid profiles compared to conventional treatments alone [180]. Other Chinese herbal medicines have also been reported to exert beneficial effects on post-PCI depression [181]. Since most of the evidence to date originates from small randomized trials conducted in China, more rigorous studies with larger sample sizes are needed to confirm the use of traditional Chinese medicine for the treatment for depression following ACS.

## 5. Conclusions

**Fig. 1.**
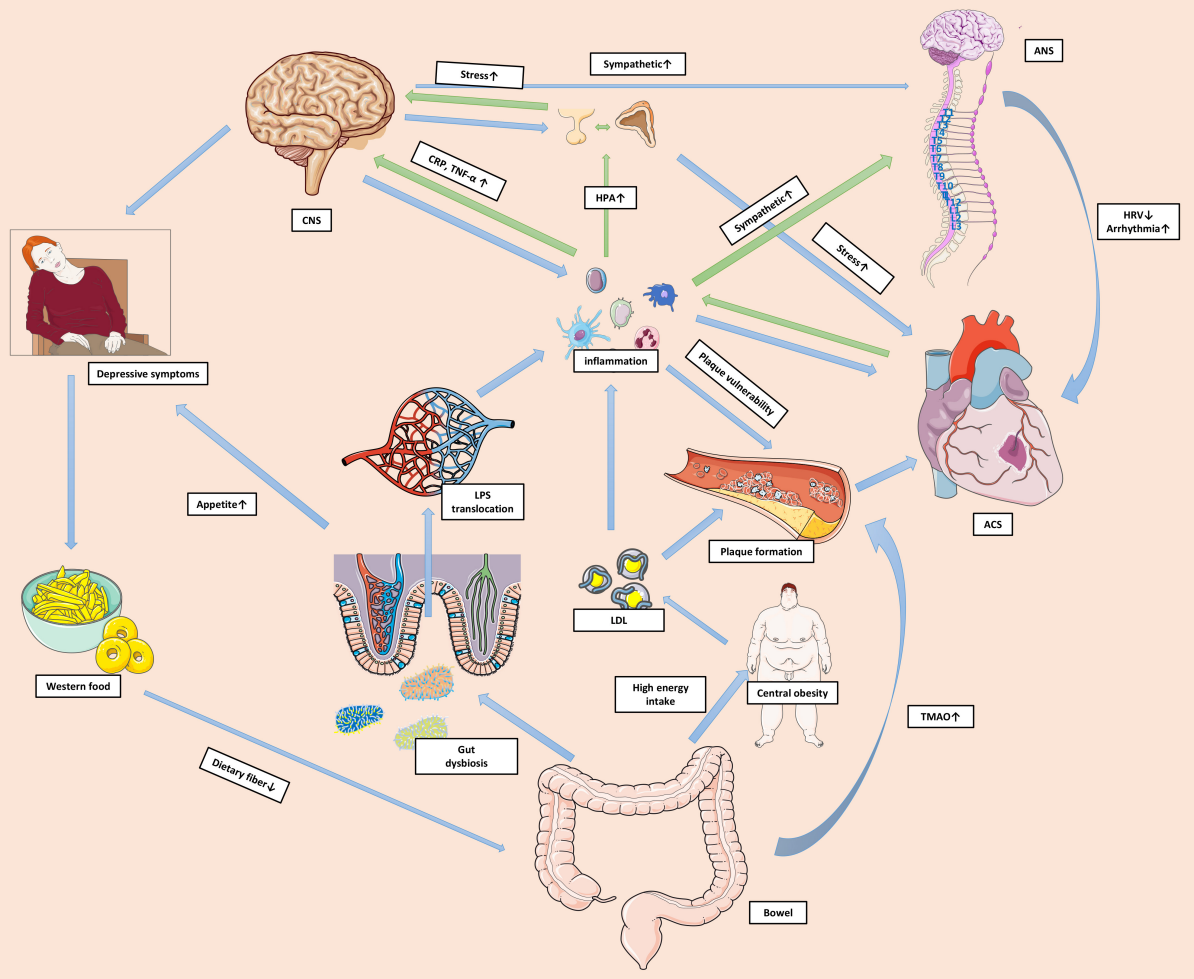
**Shared mechanisms of depression and ACS**. A shared network of biological and behavioural mechanisms contribute to the reciprocal relationship between ACS and depression. ACS prompts substantial activation of local and systemic inflammation. The unresolved systemic inflammation alters neural functions and activates the HPA axis and sympathetic system via pro-inflammatory mediators. Pro-inflammatory mediators impact CNS signaling to regulate mood and behaviours, resulting in depressive symptoms. Excessive cortisol makes the body vulnerable to acute stress and may amplify the toxic effects of environmental threats. Immune cells in the CNS are also activated and perpetuate the systemic inflammation. Increased catecholamine levels lead to lower HRV and more arrhythmic events. Inflammation also causes platelet and endothelial dysfunction, and impacts plaque formation and stability. Furthermore, depressive symptoms can manifest as excessive intake of food that is high in fat and low in dietary fiber, resulting in gut dysbiosis. This in turn can increase the permeability of the gut barrier and facilitate translocation of the pro-inflammatory factor LPS into the circulation. High energy intake can also cause central obesity and hyperlipidemia. Some harmful metabolites, such as TMAO, can accumulate and directly impact cardiac health. The green arrows in the figure represent the pathophysiological pathways from ACS to depression, while the blue arrows represent the pathways from depression to ACS. Abbreviations: ACS, acute coronary syndrome; ANS, autonomic nervous system; CNS, central nervous system; CRP, C-reactive protein; HPA, hypothalamic-pituitary-adrenal; HRV, heart rate variability; LPS, lipopolysaccharide; LDL, low-density lipoprotein; TMAO, trimethylamine N-oxide; TNF-α, tumor necrosis factor-α.

Depression is a common comorbidity that affects approximately 20% of patients with ACS. Such patients are at high risk for long-term adverse clinical outcomes, meaning that more attention should be paid to this subgroup and personalized care plans should be formulated. Depression following ACS is associated with cardiac disease severity and has similar risk factors to ACS. Thus, depression appears to be an independent risk factor for ACS. Evidence of shared biological and behavioral mechanisms for depression and ACS has emerged in recent years. These shared mechanisms may be interconnected and contribute to the reciprocal relationship between the two conditions. Fig. 1 delineates a network of biological and behavioral mechanisms involved in the pathophysiology of ACS and depression. Although the guidelines recommend screening and management of depression in patients with ACS, real world clinical practice is still far from optimal. There is a lack of evidence to support routine screening for depression. Moreover, the inconsistent results between clinical trials on the efficacy of treatment for depression in patients with ACS is a major challenge. Further studies should focus on developing more reliable and easy-to-use screening tools to improve guideline adherence. Although SSRIs appear to be safe for the treatment of depression following ACS, possible adverse outcomes and drug–drug interactions should not be ignored. Other treatments such as psychotherapy and cardiac rehabilitation appear suitable for this group of patients. Shared decision-making should be facilitated so that patients can address problems and actively engage in therapeutic programs. A multidisciplinary approach involving cardiologists, psychologists, and primary healthcare providers is essential for the successful management of complications. For patients who do not respond to treatments, a deeper insight into the underlying mechanisms and new therapeutic directions should help to solve this challenging clinical problem. With a better understanding of the mechanisms involved, the health of this patient subgroup should improve considerably.

## Abbreviations

ACS, acute coronary syndrome; AHA, American Heart Association; AMI, acute myocardial infarction; ANS, autonomic nervous system; BDI, Beck depression inventory; CABG, coronary artery bypass grafting; CAD, coronary artery disease; CBT, cognitive behavioral therapy; CHD, coronary heart disease; CRP, C-reactive protein; CVD, cardiovascular disease; ENRICHD, Enhancing Recovery in Coronary Heart Disease; FMD, flow-mediated dilatation; HPA, hypothalamic-pituitary-adrenal; HR, hazard ratio; HRV, heart rate variability; LVEF, left ventricular ejection fraction; MACE, major adverse cardiac events; MI, myocardial infarction; MIND-IT, Myocardial Infarction and Depression-Intervention Trial; OR, odds ratio; PCI, percutaneous coronary intervention; PHQ, Patient Health Questionnaire; PPT, positive psychology therapy; SSRI, selective serotonin reuptake inhibitors; SNRI, serotonin and norepinephrine reuptake inhibitor; ULF, ultra-low frequency.
